# Estimation of Industrial Emissions during Pyrolysis and Combustion of Different Wastes Using Laboratory Data

**DOI:** 10.1038/s41598-020-63807-w

**Published:** 2020-04-21

**Authors:** Juan A. Conesa, Núria Ortuño, Damià Palmer

**Affiliations:** 1 Chemical Engineering Department – Universidad de Alicante, Carretera de San Vicente del Raspeig, s/n, Alicante, 03690 Spain; 2 Industrial Engineering Department, Institut Químic de Sarrià - Universitat Ramon Llull, Via Augusta, 390, Barcelona, 08017 Spain

**Keywords:** Atmospheric chemistry, Pollution remediation, Environmental impact

## Abstract

In our lab, we have been studying the emissions of different pollutants during pyrolysis and combustion of wastes under different conditions for the last three decades. These studies have focused on the effect of temperature and presence of oxygen on the production of different pollutants. Waste decomposition has been studied in a horizontal laboratory scale reactor, but no estimate has been made of the actual emissions in a conventional thermal decomposition system. In the present study, emissions during these wastes’ thermal decomposition were estimated using Aspen HYSYS. In the simulation software, the waste composition (elemental analysis) was given as an input parameter, as well as the gas flow rate used as atmosphere during the decomposition. The emitted hydrocarbons measured in the laboratory were equated to the emission of a single compound (propylene). The simulation permitted calculating the percentage of oxygen in the emitted gas, and the pollutant emissions were then recalculated under standard conditions. The emission of dioxins and furans were estimated under different conditions of decomposition, and an adequate approximation of the waste decomposition in actual incineration systems could be obtained.

## Introduction

Thermal decomposition of wastes is considered as a valid technique to recover chemicals and/or energy contained in wastes. Uncontrolled conditions of decomposition should naturally be avoided, to ensure that the formation of emissions is controlled and to take advantage of the process. The species emitted during uncontrolled thermal degradation can lead to major health and environmental hazards.

The University of Alicante research group ‘Waste, Energy, Environment and Nanotechnology’ (WEEN) has been studying the pyrolysis and combustion of different organic wastes for the past thirty years. Initial studies were dedicated to determining the kinetics of different waste decomposition using a thermobalance. Later studies focused on the pollutants produced under different experimental conditions, using a quartz tube reactor placed inside a horizontal furnace. The atmospheres used in both types of studies were both nitrogen and synthetic air, to simulate pyrolysis and combustion conditions, at temperatures between 375–1100 °C. The studied pollutants included: carbon oxides, light hydrocarbons, polycyclic aromatic hydrocarbons (PAHs), chlorobenzenes (ClBzs), chlorophenols (ClPhs), polychlorinated dibenzo-p-dioxins and dibenzofurans (PCDD/Fs) and dioxin-like polychlorinated biphenyls (dl-PCBs). The wastes under study included: automotive shredder residue (ASR), solid recovered fuel (SRF), tyres, sewage sludges, polyvinyl chloride, polychloroprene, different fractions of electric and electronic wastes, cotton and polyester fabrics, meat and bone meals, olive oil wastes and different biomass samples, among others.

In the present study, emissions of different wastes during thermal decomposition were simulated using Aspen HYSYS. A carbon mass balance test was performed to calculate the oxygen percentage in the emitted gas, and the emitted pollutants were recalculated under normal conditions (Nm^3^). Furthermore, we examined the evolution of the H/C ratio under different conditions (temperature, presence of oxygen) to look for non-anticipated results. Emissions of PCDD/Fs were also estimated under different decomposition conditions.

Previous studies on the extrapolation of laboratory emission data to industrial scale are scarce. Ficarella and Laforgia^1^ pretend to optimize a hazardous waste incinerator in order to minimize the pollutant emission. Their approximation is to consider a high amount of reactions taking place in the decomposition chamber, and evaluating the decomposition rate of dioxins in different chamber geometries. Similarly, Bensabath *et al*.^2^ estimated the emission of polycyclic aromatic hydrocarbons (PAH) in the pyrolysis of some fuels using detailed kinetic modelling. Black *et al*.^3^ evaluated the effect of experimental methods on the emission factors for dioxins and furans emissions, concluding that field sampling and laboratory simulations were in good agreement, although they did not implement mathematical simulation.

## Experimental data

**Figure 1:**
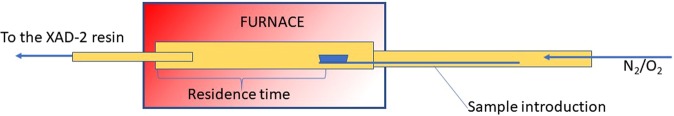
Schematic diagram of the batch laboratory scale tubular reactor used in the different studies on the decomposition of wastes.

Over the past 30 years, numerous studies have been conducted at the University of Alicante (UA) laboratory on the thermal decomposition of a large variety of wastes under different thermal degradation conditions. Over time, different ovens and waste introduction systems have been used, though a general pattern, shown in Fig. 1, has always been followed. In such systems, the waste sample is introduced at a controlled speed into an oven at a programmed temperature. The runs’ nominal temperatures varied between 375 and 1100 °C. The evolved pollutants were sampled in different ways. Analytical methods are detailed in the ‘Methods and materials’ section. Briefly, the Amberlite XAD-2 resin was inserted in the exit pipe and later extracted using solvents to analyse the various semivolatile species. Also, the gas was collected in a Tedlar bag for later analysis.

The present study comprises emission data from a total of 98 experimental runs corresponding to 20 different types of waste. In a previous paper^4^ it was evaluated the reproducibility of similar runs to that presented in this work, where it is shown that the reproducibility is quite good for all kind of compounds analysed in the emissions from pyrolysis and combustion of polyurethane foams.

In previous studies^5–17^, the evolution of the different pollutants emissions’ was analysed as a function of the experimental conditions in the decomposition zone. In addition to the temperature and residence time of the gas in the hot zone, the presence of oxygen was controlled by using a constant air flow and by modulating the rate of introduction of the waste inside the furnace. To quantify the excess (or deficiency) of air, an oxygen ratio was defined as follows (modified from Fullana *et al*.^18^):

1 $${\lambda }_{C}=\frac{{({m}_{{O}_{2}})}_{actual}}{{({m}_{{O}_{2}})}_{stoichometric}}=\frac{{m}_{air}\cdot 23+\frac{( \% O){m}_{sample}\nu }{L}}{\frac{{m}_{sample}\nu }{L}\left(\frac{ \% C}{12}+\frac{1}{4}\left( \% H-\frac{ \% Cl}{35.5}\right)+\frac{ \% S}{32}+\frac{ \% Cu}{63.5}\right)\cdot 32}$$

where:

%O, %H, %S, %C, % Cl, %Cu = weight percentage of oxygen, hydrogen, sulphur, carbon, chlorine and cupper in the waste sample; m_air_ = air flow rate (kg/s); m_sample_ = weight of the waste (kg); L = length of the tube occupied by the residue (m); ν = linear velocity of introduction of the sample in the furnace (m/s).

Using this definition, a λ_C_ value below one implies combustion under sub-stoichiometric conditions, while λ_C_ values above one represent excess air. In pyrolytic conditions, the *λ*_C_ value can be different from zero if the sample waste contains oxygen. In this case, a limited amount of oxygen can be a source of production of oxygenated compounds, particularly PCDD/Fs and related compounds^19,20^.

## Simulation runs and discussion

We calculated the different compound emissions on a weight/weight basis for each run in such a way that emitted hydrocarbons (and PAHs in some cases) were analysed and referred to the input weight of waste (emitted compound mass/waste mass in the run).

### Calculation of the average H/C emission ratio and representativeness of the propylene

To determine a representative compound of total hydrocarbon emissions, the average H/C ratio was calculated for each run, based on the following relationship:

2 $$Average\,H/C=\frac{{\sum }_{i}^{all\,analysed\,HCs\,}\left[\frac{mg\,of\,compound{\prime} \,i{\prime} }{g\,of\,waste}\cdot {(H/C)}_{i}\right]}{{\sum }_{i}^{all\,analysed\,HCs\,}\left[\frac{mg\,of\,compound{\prime} \,i{\prime} }{g\,of\,waste}\right]}$$

According to this equation, the average H/C ratio underwent a drop between 0,80 and 3,90 in all the runs considered, as will be shown later. A single compound representing output hydrocarbon emissions was propylene, as its H/C ratio was 2 and the formation of this compound was very common in most of the runs.

### Run simulations in HYSYS and calculation of the gas emission composition

**Figure 2:**
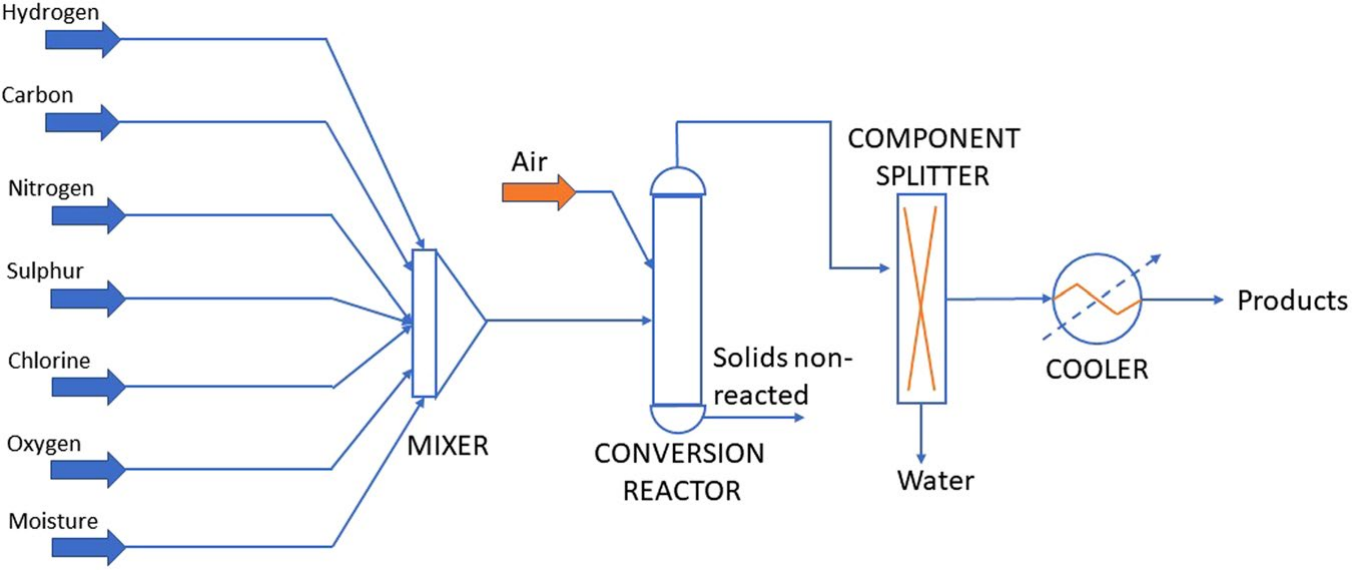
Schematic diagram of the units (in uppercase) and streams (in lowercase) used to simulate the pyrolysis and combustion runs in the Aspen HYSYS chemical process simulator.

The experimental runs were simulated using Aspen HYSYS V10 setting a Fluid Package based on the Peng-Robinson Equation of State. To simulate each experiment performed in the laboratory furnace, one current per element was added, representing the different elements composing the waste (C, H, O, N, S, Cl and moisture); later, another current with air at 1 atm was added (Fig. 2). In each run, the λ_C_ value was known based on laboratory data, together with the waste input velocity and air flowrate. The simulated currents entered a conversion reactor (set at each run’s combustion temperature) where the reaction operates on a stoichiometric basis and will run until either the limiting reagent is exhausted, or the specified conversion has been achieved. In this conversion reactor, the reactions that took place were the formation of HCl from the corresponding halogen and hydrogen, the formation of H_2_O with the rest of the hydrogen present, the formation of SO_2_ from sulphur, the oxidation of copper when present in the residue, and the formation of CO and CO_2_. Note that mass balances are solved and forced to fulfil the analytical results, and the conditions of P, V and T are calculated following the Peng Robinson equation of state, being all calculations integrated in the HYSYS tool. The mass balance is fitted to the experimental data manually, so that to the gas stream the consumed O_2_ is eliminated and the generated CO_2_, CO and COT are added.

The production of the different hydrocarbons was also modelled using only one compound: propylene; it enabled obtaining an adequate approximation of the average composition of the hydrocarbons produced, as mentioned before. During the modelling, we assessed the conversion of the different reactions to match the final production of the known species with that obtained experimentally.

The reactor’s bottom stream contains all the solid products that can be produced, such as Sulphur or Carbon excess that does not react during pyrolysis or combustions with a low O_2_ ratio. The model includes a component splitter, coming after the reactor, that eliminates water vapour without producing any changes to other properties. The final current is then cooled at 25 °C (normal conditions). The oxygen’s molar fraction is then obtained, and the normalised flowrate can be calculated based on this last current, by using the following relationship^21^:

3 $$Normalized\,gas\,flowrate\left(\frac{N{m}^{3}}{h}\right)=Output\,gas\,flowrate\left(\frac{N{m}^{3}}{h}\right)\,\ast \,\frac{21-11}{21- \% O\,in\,the\,output\,gas}$$

In the previous equation, the oxygen percentage under normal conditions was established at 11%. Following this procedure, the different simulation runs performed in the laboratory allowed calculating the emissions on a normalised standard basis and then estimating the emissions of industrial-scale equipment.

Using this model, it is possible to estimate the total gas flow rate produced as well as its oxygen content. As an example, Table 1 shows the calculations conducted during the decomposition modelling of automotive shredder residue (ASR) waste. Following this procedure, it is possible to calculate the normalised flow rate (25 °C, 1 atm, 11% O_2_) and then to estimate the corresponding emissions based on industrial equipment.

**Table 1:** Conditions of the runs, measured parameters used for simulation of ASR decomposition and calculated emission levels.

	Temperature (°C)	600	600	600	600	600	850	850	850	850	850
INPUT VARIABLES	λ_C_	1,54	0,96	0,59	0,39	0,0642	1,52	0,98	0,58	0,36	0,0642
	Sample mass (g)	1,080	0,880	1,010	0,970	1,050	1,080	0,870	1,020	1,030	1,040
	Feed rate (mm/s)	0,2	0,4	0,6	1,0	1,0	0,2	0,4	0,6	1,0	1,0
	Sample pan length (mm)	290	290	290	290	290	290	290	290	290	290
	Air flowrate (mL/min)	500	500	500	500	(Nitrogen)	500	500	500	500	(Nitrogen)
	CO_2_ (mg/kg)	186200	352700	419800	515500	108700	382000	641700	505800	470300	86900
	CO (mg/kg)	69500	140200	125000	119600	37300	2700	64300	211800	284500	94600
	Total Organic Carbon (mg/kg)	13370	28952	77821	75590	165623	16	9044	10375	135680	259592
	PCDD/Fs (pg I-TEQ/g)	5440,00	28500,00	7720,00	7110,00	309,00	81,00	50,40	29,70	77,20	22,50
CALCULATED VALUES	Sample feed rate (kg/h)	2,68E-03	4,37E-03	7,52E-03	1,20E-02	1,30E-02	2,68E-03	4,32E-03	7,60E-03	1,28E-02	1,29E-02
	CO_2_ emission (g/h)	0,49927	1,54118	3,15805	6,20733	1,41685	1,02429	2,77214	3,84268	6,01335	1,12191
	CO emission (g/h)	0,18636	0,61263	0,94034	1,44015	0,48619	0,00724	0,27778	1,60910	3,63768	1,22132
	Propylene emission (g/h)	0,03585	0,12651	0,58543	0,91020	2,15880	0,00004	0,03907	0,07882	1,73483	3,35142
	STD gas flow (Nm^3^/h)	0,029	0,029	0,029	0,031	0,007	0,029	0,029	0,028	0,031	0,010
	O_2_% output current	17,22	12,82	7,58	0,00	0,00	16,54	11,31	4,99	0,01	0,00
	CO_2_ (mg/Nm^3^)	17068	53279	108914	200946	214865	35112	96292	137360	194923	110364
	CO (mg/Nm^3^)	6371	21179	32430	46621	73730	248	9649	57518	117915	120143
	Propylene (mg/Nm^3^)	1225	4374	20190	29465	327383	1	1357	2818	56235	329685
	PCDD/Fs (ng I-TEQ/Nm^3^)	498,00	4310,00	2000,00	2770,00	610,00	7,45	7,56	8,06	32,00	28,60

The operation is as follows:

4 $$\begin{array}{c}PCDD/Fsemission\left(\frac{pg\,I-TEQ}{N{m}^{3}}\,\right)=PCDD/Fs\,emission\,factor\left(\frac{pg\,I-TEQ}{{g}_{sample}}\right)\\ \,\ast \,Sample\,flowrate\left(\frac{{g}_{sample}}{h}\right)\ast {\left[Normalizedgasflowrate\left(\frac{N{m}^{3}}{h}\right)\right]}^{-1}\end{array}$$

The key point in this calculation is to check whether the legal limit (0,1 ng ITEQ/Nm^3^ of the EU^21^ and the 0,5 ng TEQ/Nm^3^ of the Chinese emissions standards^22^) is exceeded or not.

### Comparison of different waste emissions. evolution of H/C ratio vs. temperature and oxygen ratio

**Figure 3:**
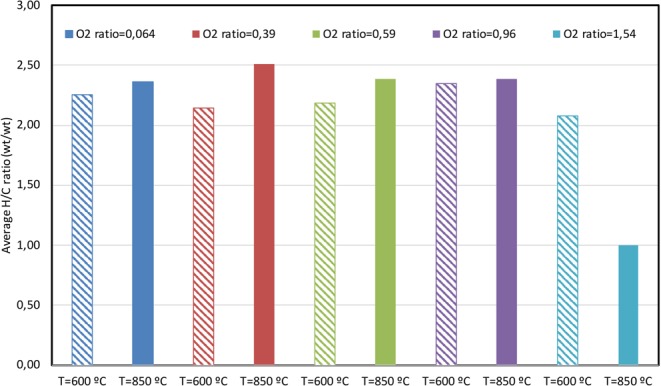
Evolution of the H/C ratio during ASR decomposition at different temperatures and oxygen ratio.

**Figure 4:**
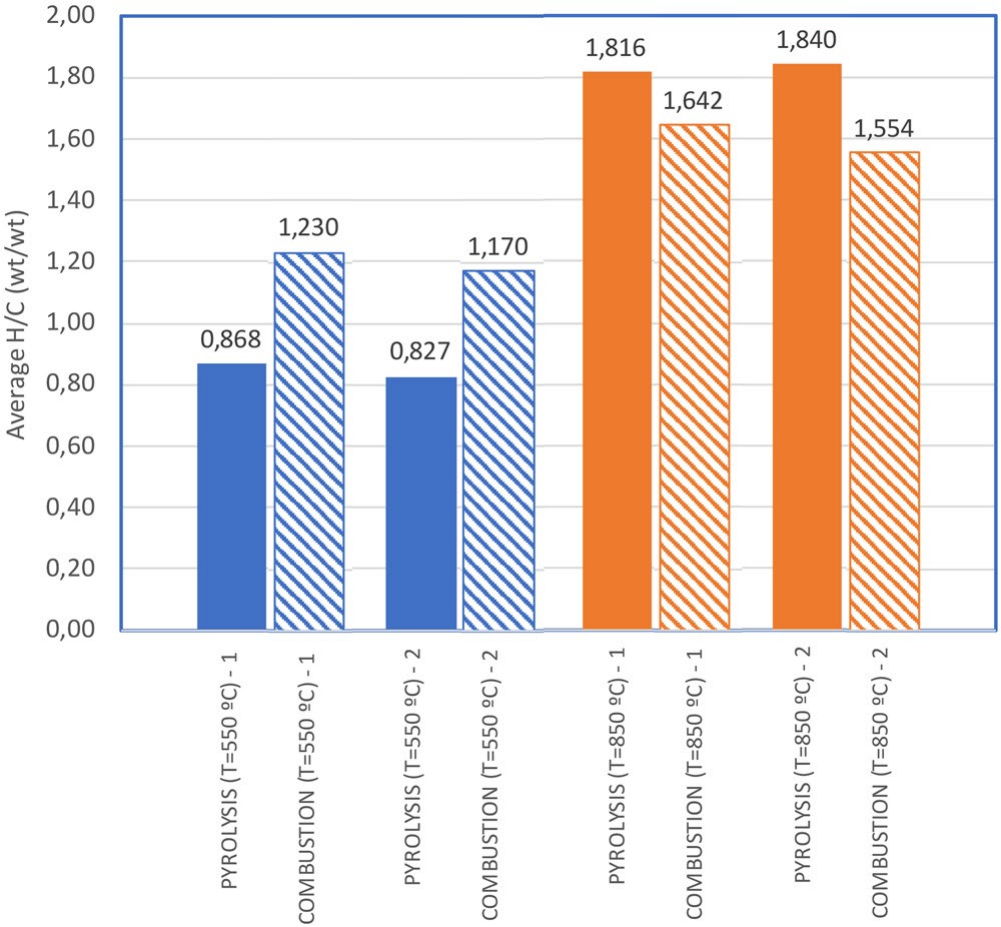
Average H/C ratio during the pyrolysis (λ_C_ = 0,11) and combustion (λ_C_ = 0,84) of flexible polyurethane foams (FPUF) at two different temperatures (550 °C and 850 °C). Duplicated runs.

**Figure 5:**
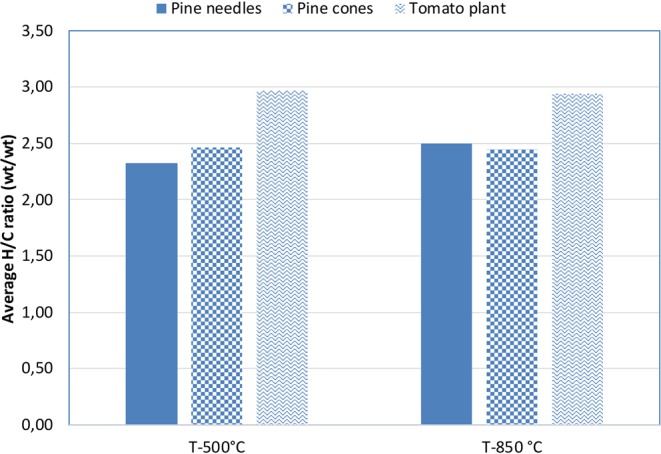
Evolution of the average H/C ratio in biomass feedstock decomposition at temperatures with λ_C_ close to 0,4.

The different wastes’ decomposition produced a variety of compounds that depended on each run’s particular conditions, specifically on oxygen excess and temperature. An average H/C ratio could be calculated for each performed run, with the aim of analysing the general behaviour and finding a compound that is representative of the total emissions. The average H/C was calculated, and some results are shown in Figs. 3–5.

Table 2 shows the C/H ratio of the starting waste materials, in order to compare them with the emissions (Figs. 3–5). The C/H ratio of the wastes is in the range 0,09–0,24, whereas in general the ratio of the emissions is much higher. This Table also shows some data on PCDD/Fs emission that will be discussed later.

**Table 2:** Different wastes studied, the C/H ratio of the starting waste material, experimental conditions (temperature and oxygen ratio), and comparison of the PCDD/F emissions between the data obtained from the laboratory experiments (pg I-TEQ/g) and the data calculated using the total flowrate correlation (ng I-TEQ/Nm^3^).

Material waste	H/C (wt/wt) of the waste	Temperature (°C)	Oxygen ratio (λ_C)_	Total PCDD/Fs (pg I-TEQ/g)	Emission (ng I-TEQ/Nm^3^) using correlation for total flowrate
MBM	0,16	700	0,07	90,40	180,00
MBM		700	0,07	7,65	15,30
MBM		850	0,07	20,10	40,00
MBM		950	0,07	6,61	13,20
MBM		975	0,07	0,77	1,54
MBM		1100	0,07	0,45	0,90
MBM		1100	0,07	2,94	5,86
MBM		600	0,65	5,52	1,19
MBM		700	0,67	27,80	5,80
MBM		700	0,82	57,40	9,76
MBM		850	0,84	1,59	0,30
MBM		950	0,72	1,05	0,20
MBM		975	0,71	4,97	1,00
MBM		1100	0,78	4,95	0,90
MBM		1000	0,47	106,00	31,40
MBM		600	0,07	5,89	11,70
MBM		850	0,07	25,10	50,00
MBM		1000	0,07	32,70	65,20
MBM		600	1,55	8,58	0,80
MBM		850	1,51	26,60	2,46
MBM		1000	1,73	151,00	12,20
PVC	0,13	850	0,0006	215,00	50020,00
PVC		850	0,089	4580,00	7180,00
PVC		375	0,46	183,00	55,50
PVC		850	1,00	224000,00	31300,00
Polychloroprene	0,11	850	0,71	11400,00	2240,00
Polychloroprene		850	0,0365	22,20	84,90
Polychloroprene		850	0,0365	23,40	89,30
Polychloroprene		850	0,71	11400,00	2240,00
Cotton Fabrics	0,15	850	0,70	8,36	1,67
Polyester fabrics	0,07	850	0,71	15,70	3,09
Sewage Sludge	0,16	850	0,57	1720,00	421,00
Sewage Sludge		850	0,57	58,50	14,30
Sewage Sludge		850	0,57	31,70	7,77
Sewage Sludge		850	0,57	35,70	8,74
Sewage Sludge		850	0,57	19,80	4,84
Sewage Sludge		850	0,44	33,50	10,60
Sewage Sludge		850	0,57	9,55	2,34
Sewage Sludge		850	0,73	73,00	14,00
Sewage Sludge		850	0,88	12,30	1,96
Sewage Sludge		850	1,20	4,59	0,50
Electronic waste	0,09	500	0,23	7,33	4,45
Electronic circuits		500	0,58	3,41	0,80
Electronic waste		500	0,045	29,10	90,20
Electronic circuits		500	0,39	28,20	10,10
Electronic Waste		850	0,23	76,40	46,40
Electronic Circuit		850	0,54	165,00	42,60
Electronic Waste		850	0,045	76,40	237,00
Electronic Circuit		850	0,39	23,50	8,42
Mobile Case	0,09	500	0,18	867,00	672,00
Mobile Case		850	0,18	17,70	13,70
Mobile Case		500	0,02	6,11	42,70
Mobile Case		850	0,02	6,11	42,70
Halogen free wire	0,24	700	0,50	71,00	19,80
Halogen free wire		700	0,50	66,90	18,70
PVC wire	0,16	700	0,50	6940,00	1940,00
PVC wire		700	0,50	41300,00	11500,00
SRF	0,13	850	1,45	777,00	74,80
SRF		850	1,00	2790,00	390,00
SRF		850	0,66	301,00	63,60
SRF		850	0,49	117,00	33,30
ASR	0,13	600	0,0642	309,00	673,00
ASR		600	0,39	7110,00	2540,00
ASR		600	0,59	7720,00	1830,00
ASR		600	0,96	28500,00	4150,00
ASR		600	1,54	5440,00	493,00
ASR		850	0,0642	22,50	48,90
ASR		850	0,36	76,90	29,80
ASR		850	0,58	29,70	7,14
ASR		850	0,98	50,40	7,17
ASR		850	1,52	81,00	7,44
FPUF	0,12	550	0,1103	6,80	8,60
FPUF		550	0,1103	30,70	38,80
FPUF		850	0,1103	18,50	23,50
FPUF		850	0,1103	28,60	36,10
FPUF		550	0,85	65,10	10,70
FPUF		550	0,85	60,70	10,00
FPUF		850	0,85	62,90	10,30
FPUF		850	0,85	96,60	15,90
VMF	0,05	550	0,1158	2,86	3,45
VMF		650	0,1158	3,78	4,55
VMF		750	0,1158	5,39	6,50
VMF		850	0,1158	0,20	0,20
VMF		550	0,82	0,55	0,00
VMF		650	0,82	3,41	0,60
VMF		750	0,82	3,48	0,60
VMF		850	0,82	20,30	3,45
VMF		850	0,82	17,70	3,01
Furniture Wood Waste	0,13	850	0,39	21,00	7,50
Furniture Wood Waste		850	0,39	15,20	5,45
Furniture Wood Waste		850	0,39	20,10	7,20
Solid Wood	0,12	850	0,39	1,79	0,60
Pine Needles & Cones	0,13	850	0,36	54,50	21,10
Pine Needles & Cones		850	0,35	601,00	240,00
Pine Needles & Cones		850	0,36	50,40	19,60
Pine Needles & Cones		850	0,35	98,20	39,10
Tomato Plant	0,14	500	0,46	923,00	280,00
Tomato Plant		850	0,46	147,00	44,60

MBM: meat and bone meal.

PVC: poly vinyl chloride.

SRF: solid recovered fuel.

FPUF: flexible polyurethane foam.

ASR: automotive shredder residue.

VMF: viscoelastic memory foam.

Figure 3 shows the evolution of the H/C ratio during the ASR decomposition. The H/C ratio for this waste is 0,13, and much higher in the gases emitted at the different conditions, due to the reaction of carbon to give carbon oxides, among others. The increase in temperature (from 600 °C to 850 °C) produced an increase in the H/C ratio of the emitted gases for runs performed with oxygen ratio values below 1. The greatest presence of oxygen in the last case (see Fig. 3, bars corresponding to an O_2_ ratio = 1,54), however, substantially decreased the H/C ratio. This is due to the oxygen’s reaction with the different hydrocarbons, especially with lesser stable ones such as those with higher H/C, i.e. alkanes. On the other hand, the H/C ratio of hydrocarbon emissions at the different oxygen ratios was more or less similar when λ_C_ < 1, both at 600 °C and 850 °C. In this sense, the H/C ratio was somewhat constant at a temperature of λ_C_ < 1 but the excess oxygen caused H/C to clearly drop when going from 600 °C to 850 °C. It is worth noting that the average H/C ratio does not represent the total amount of emitted compounds, which naturally decrease as oxygen presence increases^23,24^.

During the ASR decomposition, the most abundant hydrocarbons were methane, ethylene and propylene for both pyrolysis and combustion runs. In the absence of oxygen, light hydrocarbons showed higher yields, indicating that the latter are easily oxidised in combustion experiments^16^. As the temperature increases, most hydrocarbons decrease their yields, except in the case of runs under pyrolytic conditions.

The H/C ratio increase with rising temperatures was also observed for other wastes. Figure 4 shows the evolution of FPUF waste decomposition. In the case of this material, runs were conducted under pyrolytic conditions (λ_C_ = 0,11) and with a moderate presence of oxygen (λ_C_ = 0,84). Duplicate runs were performed under all experimental conditions and they produced very similar results. As shown in Fig. 4, the H/C ratio increased from 0,82 (at 550 °C) to 1,81 (at 850 °C) in pyrolysis experiments, and from 1,23 to 1,64 in the presence of oxygen. Pyrolytic conditions produced similar H/C ratios to that of combustion runs; this is unsurprising given that the combustion experiments were carried out under highly fuel-rich conditions. As mentioned before, a higher oxygen ratio value would lead to a drop in the H/C ratio.

Many different wastes have been studied in recent years, including biomass feedstock decomposition: pine needle and cone, as well as tomato plant decomposition were studied in detail in recent studies^25,26^. The average H/C ratio was calculated based on the data presented in these papers, and the results are shown in Fig. 5. In this case, the λ_C_ value ranged between 0,35 and 0,46. For these materials, the average H/C ratio was relatively high compared to other wastes, indicating the presence of more saturated hydrocarbons, particularly methane, in the gas emissions. The evolution with rising temperature shows a similar behaviour to that mentioned previously.

When conducting a similar analysis for the different wastes under study, the average H/C ratio ranged between 0,80–3,90. To simulate the evolved hydrocarbons, a single compound representing the output hydrocarbon emissions was taken into account. It is possible to make a proper estimate using propylene, because its H/C ratio is 2, and large amounts of this compound can usually be found in gas emissions.

### PCDD/F emissions from different wastes. estimation of industrial emissions

**Figure 6:**
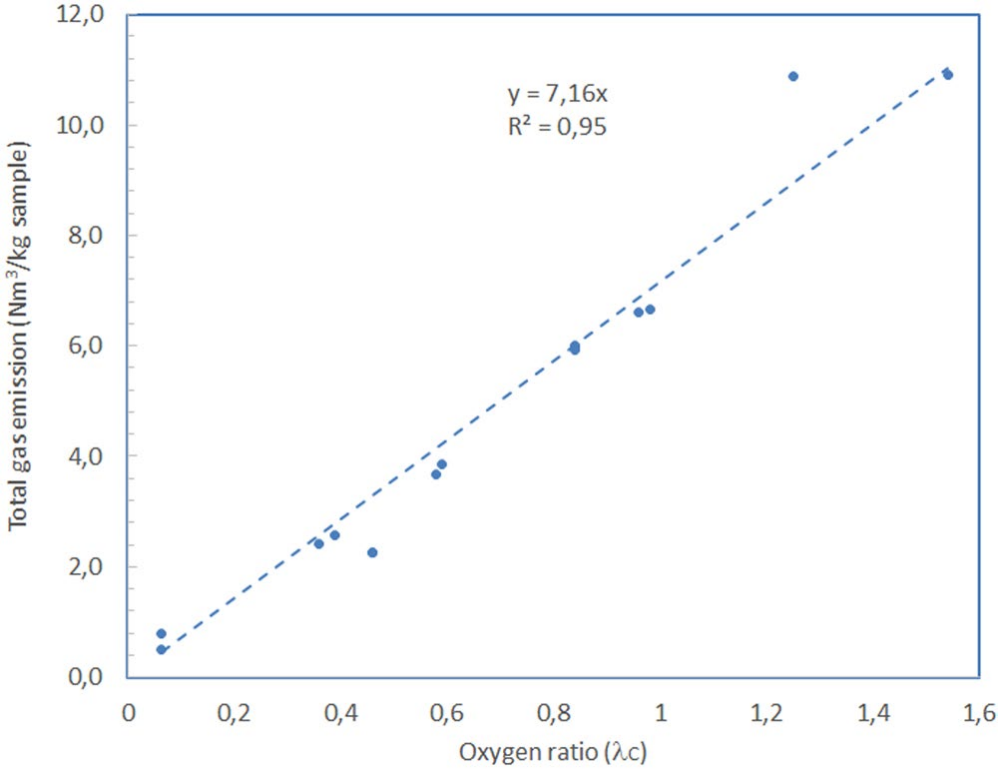
Correlation between the oxygen ratio used in the runs and the total calculated gas emissions.

Though one may consider it a very rough rule, a correlation exists between the values of the total calculated amount of gas and the introduced oxygen ratio. Figure 6 shows this correlation, which can be modelled by the following equation:

5 $$\frac{N{m}^{3}gas}{k{g}_{sample}}=7,1566\cdot {\lambda }_{C}$$

In this way, the following relationship could be derived:

6 $$PCDD/F\,emission\,\left(\frac{pg\,I-TEQ}{N{m}^{3}}\,\right)=\frac{PCDD/F\,emission\,factor\,\left(\frac{pg\,I-TEQ}{{g}_{sample}}\right)}{7,1566\cdot {10}^{-3}\cdot {\lambda }_{C}\left(\frac{N{m}^{3}}{{g}_{sample}}\right)}$$

The emission levels calculated using the model (shown in Table 2) were, in most cases, above the legal limit. This means that thermal decomposition under experimental conditions would (sometimes) produce a very high level of pollutants, in some cases exceeding 2500 ng I-TEQ/Nm^3^. This result was expected, as the decomposition processes were carried out under conditions of poor oxygen presence (fuel-rich combustions), that maximise the formation of incomplete combustion products. Nevertheless, the model allows calculating the emissions of industrial incineration/gasification equipment under optimised operating conditions.

A study presenting actual emission of PCCD/Fs in municipal solid waste (MSW) incinerators^27^ shows that the emission are usually in the range 0,5–31,8 ng ITEQ/Nm^3^. This MSW is similar to SRF, both coming from household waste. Table 2 shows that the emission for SRF decomposition in oxygen-rich conditions (λ_C_ = 1,45) is 74,8 ng ITEQ/Nm^3^.

However, emissions under pyrolytic conditions should not be compared with proposals within legal limits, since these limits refer mainly to combustion conditions with excess air. During pyrolytic runs the amount of oxygen in the reaction atmosphere is almost zero (as it only comes from the oxygen in the waste itself). For this reason, the extrapolation of the composition of the gases obtained in pyrolytic runs to the standard 11% O_2_ can be difficult.

In previous studies^20^, the upper limit of industrial equipment emissions was estimated based on an average volume of evolved gases and a particular waste feed rate. In the present study, the emitted pollutants under the different oven conditions could be easily extrapolated to larger equipment, maintaining the decomposition conditions.

### PCDD/F emissions from different wastes. comparison of wastes

**Figure 7:**
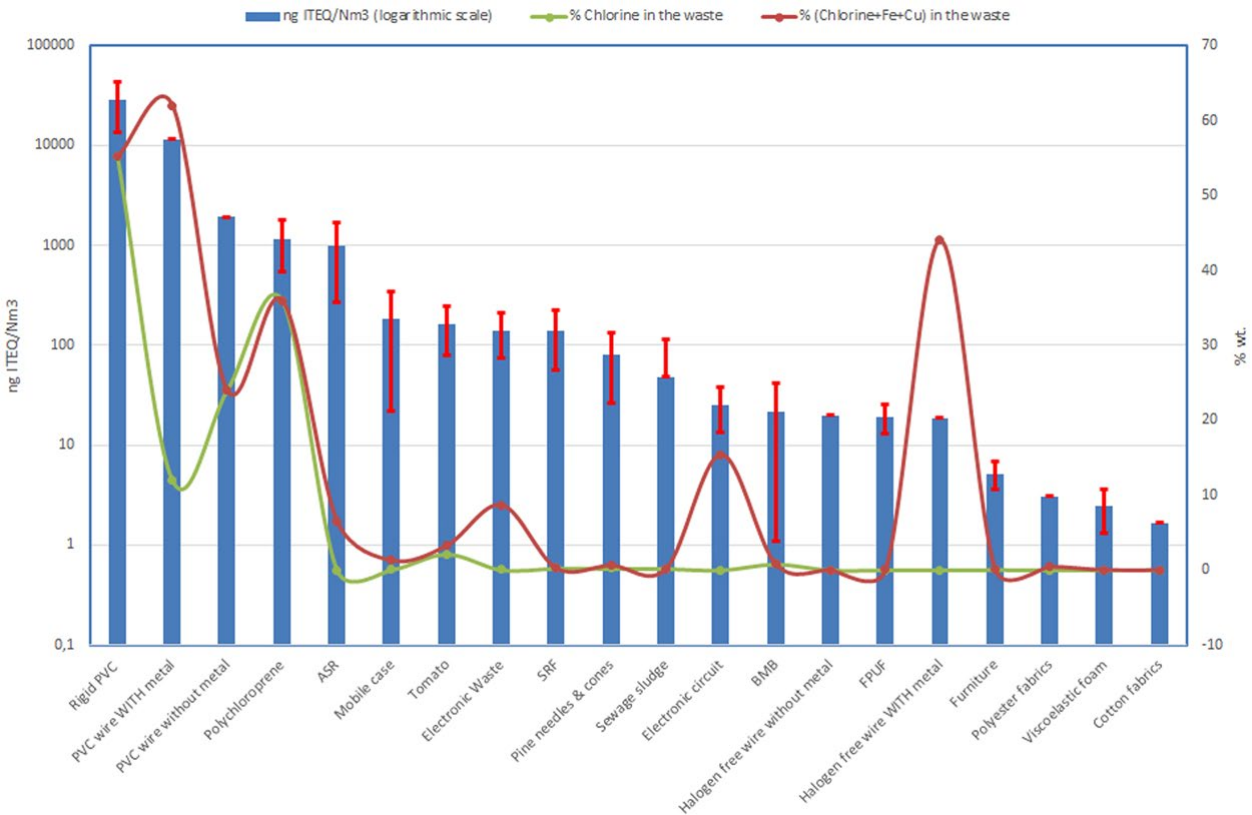
Emissions of PCDD/Fs from the thermal decomposition of different wastes (average of runs performed under different conditions, with standard deviation) and chlorine and metal content in the waste samples.

Using all the data previously published by our research group, we also calculated average PCDD/Fs emissions during the thermal decomposition of all wastes used, without distinguishing decomposition conditions (presence of air, temperature). The aim was to estimate which wastes tended to produce more dioxins and furans during their primary decomposition. The results are shown in Fig. 7, on a logarithmic scale, as the values were quite different across the wide range of wastes tested. Figure 7 also shows the percentage of chlorine present in the waste, and the percentage of the sum of chlorine, iron and copper, some of the major catalysts of PCDD/Fs formation (Chlorine + Fe + Cu). The amount of emitted PCDD/Fs can be seen to closely correlate with the presence of chlorine. A few notable exceptions, however, are worth mentioning. The decomposition of PVC wire in the presence of metals (mainly Cu) produced a substantial amount of dioxins, which was much higher compared to when no metal was present. This points to the fact that the presence of metal catalyses the formation of these pollutants. The presence of metal was also high in the “Electronic circuit” and “Halogen free wire with metal” samples. Low emissions in both cases indicated that the presence of metal alone was insufficient to produce high amounts of dioxins: a certain percentage of chlorine in the waste is also needed. This result is compatible with previous literature that examined the role of the presence of metals and chlorine in the production of PCDD/Fs^28–32^.

It is worth noting that the data in Fig. 7 represent the dioxin and furan content in the gas emitted during the primary decomposition, with no influence of the post-combustion process usually present in the incinerators, and obviously with no presence of air pollution control devices. Emissions from a real incineration plant would be affected by both processes, greatly diminishing the pollutants emitted.

Furthermore, in recent years, abundant research has been conducted on the ability of certain N and S containing compounds to prevent the formation of PCDD/Fs during the thermal destruction of wastes. Thiourea, ammonia thiosulphate and sulfamic acid have been extensively studied. Different authors^33,34^ describe a drop of over 95% in emissions of chlorinated compounds when these compounds are present.

## Methods and materials

Gases and volatile compounds were analysed by gas chromatography coupled to different detectors: the CO_2_ and CO was quantified using a thermal conductivity detector (GC-TCD, model Shimadzu GC-14A, fitted with an Alltech CTR I column) while light hydrocarbons (C_1_–C_6_) together with benzene, toluene and xylenes were analysed using a flame ionization detector (GC-FID, model Shimadzu GC-17A, fitted with an Alumina KCl Plot capillary column). Six different gas standard mixes containing known amounts of hydrocarbons C_1_–C_6_, CO_2_ and CO, with the balance completed with N_2_, were used to calibrate the gas chromatographs.

To analyse the organic semivolatile compounds, the sampling resin was Soxhlet extracted with dichloromethane in accordance with the US EPA method 3540C^35^ or by means of accelerated solvent extraction (Dionex ASE 100) with dichloromethane/acetone (1:1 vol.) according to U.S. EPA method 3545A^36^. The extracts had a concentration of approximately 1 mL and a recovery standard was added. For the 16 priority PAH analysis^37^, deuterated internal standards were added to the resin at the beginning of the process and the extracts were analysed by HRGC-MS (Agilent 6890N GC coupled to an Agilent 5973N MS) with an Agilent HP5-MS capillary column (30 m × 0,25 mm i.d. × 0.25 μm) in accordance with the U.S. EPA method 8270D^38^. These compounds were analysed in the SCAN mode (35–550 amu) with native standards used to identify and quantify them, whereas other semivolatile compounds were identified by comparison with the NIST mass spectral library, interpolating between the response factors from the two nearest deuterated standards for semi-quantification.

The PCDD/Fs analysis was performed using a HRGC (Agilent HP5890) coupled to a HRMS (Waters Micromass Autospec Ultima NT) in positive electron impact (EI+) mode. The analytical procedure consisted in extraction with toluene, solvent change solvent to n-hexane, acid treatment with sulphuric acid when necessary, and clean-up using the Power Prep^TM^ system (FMS Inc., USA) with three different columns: silica, alumina and activated carbon (FMS Inc., USA). The analyses were carried out in compliance with the U.S. EPA method 1613^39^.

All solvents for organic trace analysis were purchased from Merck, Germany (dichloromethane, acetone, toluene, n-hexane, ethyl acetate and nonane) and were pesticide grade. Standards were supplied by Dr. Ehrenstorfer, Germany (PAH Mix 63 and Internal Standards Mix 26 for PAHs), Wellington Laboratories, Canada (EPA-1613 solutions for PCDD/Fs) and AccuStandard, USA (anthracene-d_10_, used as recovery standard).

Laboratory blanks (without sample) were carried out before each set of combustion or pyrolysis experiments using the laboratory scale reactor under the same conditions as the runs. A complete and interesting dataset was collected from these series of runs, that combined different wastes and conditions of thermal decomposition (temperature, residence time, oxygen presence). Specifically, data from the following previous studies were used in the present work (classified according to the waste used in the study):

- Meat and bone meal (MBM)^5^
- Poly vinyl chloride (PVC)^6^
- Cotton and polyester fabrics^7,8^
- Sewage sludges^9,10,19^
- Electronic waste (including materials from mobile phones and electric wires)^11–13^
- Polychloroprene (neoprene)^14^
- Solid Recovered Fuel (SRF)^20^
- Mattresses wastes (viscoelastic and polyurethane foams)^4,15^
- Furniture wood waste^17^
- Automotive Shredder Residue (ASR)^16^
- Pine cones and needles^26^
- Tomato plant^25^.

Table 1 shows the calculations conducted during the decomposition modelling of one particular ASR waste. Similar calculations were applied to the rest of the wastes under study.

Table 2 shows a summary of the results of the decomposition of several studied wastes under specific experimental conditions and PCDD/F emission values, both experimental and calculated.

## Data Availability

The datasets generated and/or analysed in the current study are available from the corresponding author upon reasonable request.
